# Flexible Visuomotor Associations in Touchscreen Control

**DOI:** 10.3389/fnhum.2017.00558

**Published:** 2017-11-17

**Authors:** Sara Fabbri, Luc P. J. Selen, Robert J. van Beers, W. P. Medendorp

**Affiliations:** ^1^Donders Institute for Brain, Cognition and Behaviour, Radboud University, Nijmegen, Netherlands; ^2^Department of Human Movement Sciences, Vrije Universiteit Amsterdam, Amsterdam, Netherlands

**Keywords:** visuomotor associations, swiping, spatial mismatch touchscreen control, principal component analysis (PCA), reaching

## Abstract

To move real objects, our hand needs to get in direct physical contact with the object. However, this is not necessarily the case when interacting with virtual objects, for example when displacing objects on tablets by swipe movements. Here, we performed two experiments to study the behavioral strategies of these movements, examining how visual information about the virtual object is mapped into a swipe that moves the object into a goal location. In the first experiment, we investigated how swiping behavior depends on whether objects were located within or outside the swiping workspace. Results show that participants do not start the swipe movement by placing their finger on the virtual object, as they do when reaching to real objects, but rather keep a systematic distance between the object location and the initial swipe location. This mismatch, which was experimentally imposed by placing the object outside the workspace, also occurred when the object was within the workspace. In the second experiment, we investigated which factors determine this mismatch by systematically manipulating the initial hand location, the location of the object and the location of the goal. Dimensionality reduction of the data showed that three factors are taken into account when participants choose the initial swipe location: the expected total movement distance, the distance between their finger on the screen and the object, and a preference not to cover the object. The weight given to each factor differed among individuals. These results delineate, for the first time, the flexibility of visuomotor associations in the virtual world.

## Introduction

Interacting with objects is a fundamental characteristic of human behavior. We unscrew lids from jars, catch balls or play the piano. But with the advent of touchscreen technology, new means of virtual interactions have emerged. We swipe across the screen to bring objects or icons in vision that are initially not seen, or swipe to rotate virtual 3D-objects, like globes or cars, to view them from other perspectives. While these types of interactions feel straightforward and intuitive, they evoke new computational challenges compared to the manipulation of real objects.

When manipulating real objects we can directly transform the perceived spatial information about the object into the corresponding motor coordinates that guide the muscular contractions (see for reviews Brenner and Smeets, 2003; Crawford et al., 2011). It is thought that in this process a movement vector is computed: i.e., the difference vector between the initial location of the effector and the location of the object (Vindras and Viviani, 1998). Behavioral signatures of the computation of this movement vector are seen in the errors of reaching movements, which depend on initial hand location, object location and eye position (Vindras and Viviani, 1998; Vindras et al., 1998; Beurze et al., 2006). Based on these observations, it has been suggested that the difference vector is represented in multiple reference frames (Beurze et al., 2006; McGuire and Sabes, 2009), which finds support in neurophysiological studies (Buneo et al., 2002; Pesaran et al., 2006).

While the mapping between the perceived location of an object and the final position of the effector used to manipulate the object is typically fixed in the real world, this mapping is flexible when we interact with virtual objects on a touchscreen. In other words, when swiping on touchscreens there is more than one way to control an object. Not only can we position our finger on the object and drag it, as we would do to move objects in the real world, we can also move an object by starting the swipe from another location on the touchscreen. For example, when we explore a map on our smartphone, we swipe to bring objects in vision that are not yet seen. Therefore, the location where we touch the screen to start the swipe can be spatially different from the location of the object to move. This imposes a flexible relationship between the object and the finger location, which means that the brain needs to resolve these additional degrees of freedom when planning and implementing an action. A further difference between action on virtual vs. real objects is the lack of object dynamics and interaction forces.

In classic experimental paradigms, the flexible association between the visual stimulus and the motor response is typically investigated by applying a contextual rule to the visuomotor mapping, e.g., a spatial rule in the anti-reach task (Medendorp et al., 2005; Gail and Andersen, 2006; Gail et al., 2009), or an arbitrary visual rule (Grol et al., 2009; Yamagata et al., 2009). However, in touchscreen control the visuomotor mapping is not rule-based, but freely chosen by the participant. This flexible mapping bears resemblance with how humans use tools, but without the effects of object dynamics or interaction forces. For example, we can hold the handle of a hammer in many ways, but still aim the head at the nail (Umiltà et al., 2008; Cattaneo et al., 2013).

As a result, the investigation of motor behavior on touchscreens offers a unique opportunity to explore the flexibility of visuomotor transformations in ecologically novel conditions.

Here we measured the kinematics of swiping movements while participants had to move an object from its initial location to a goal location on a touchscreen. In two experiments, we tested how the visuomotor mapping of swiping movements varied for flexible vs. instructed conditions (Experiment 1) and which spatial variables (i.e., location of the object on the screen, initial hand location and goal location) are used as a reference for the swiping behavior (Experiment 2). We discuss a model, which allows us to interpret which criteria participants used to choose the initial swipe location.

## Materials and Methods

### Participants

Thirty nine participants (average age 21 years, 34 female) took part in the study. All participants were right-handed and had normal or corrected-to-normal vision. They gave written informed consent to take part in the study and received student credits (1 credit/h) or monetary compensation (€10/h). The study was approved by the ethics committee of the Faculty of Social Sciences of Radboud University, Nijmegen, Netherlands.

### Apparatus and Setup

Participants sat in front of a horizontally placed touchscreen (Iiyama ProLite T2735MSC-B1 width 67.3 cm, depth 41.9 cm, 60 Hz refresh rate, reaction time 5 ms), centered with their body midline. Participants were asked to execute swiping movements on the screen using their right index finger. The experiment was programmed in PsychoPy v1.82.01 (Peirce, 2008) and presented the following items on a gray background: a 3 × 3 cm red square (“start”) indicating the required initial hand location, the “object”, a white dot of 1 cm radius and the “goal”, a green dot of 1 or 2 cm radius for the small and large goal respectively. Once the participant touched the start, the object and goal appeared on the screen, while the start disappeared. Participants were instructed to leave the start and bring the object into the goal by swiping across the touchscreen as fast and as accurate as possible, but with an upper limit of 2 s from presentation of the object to moving the object into the goal. To move the object into the goal, participants had to lift their finger from the start, and position it on the screen where they chose to initiate the swiping movement to drag the object into the goal. The object followed the same kinematics as the finger motion. The trial was considered successful once the object center was within the goal area. The trial finished either if the object was successfully dragged into the goal or when the trial timed out after 2 s had elapsed since the appearance of the object (i.e., error trial). The time between the appearance of the object and the arrival of the object in the goal was called Movement Time (MT). After a successful trial, the MT was shown on the screen. Participants were instructed to try to keep MT as low as possible. If the target was not into the goal at the end of the trial, the error message “Too slow trial” appeared on the screen. After a 1 s inter-trial interval, the next trial started.

### Experiment 1: Flexible vs. Instructed Swiping Movements

**Figure 1:**
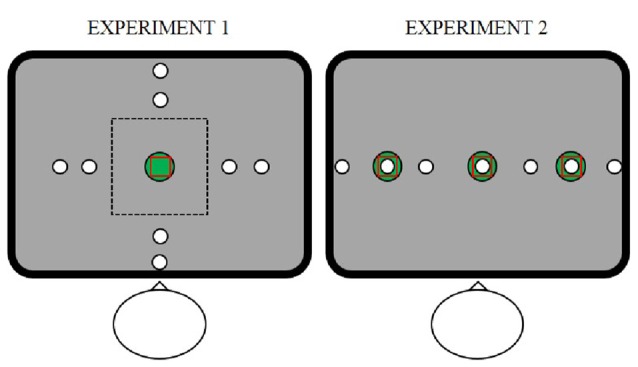
Stimulus display of Experiment 1 (left panel) and Experiment 2 (right panel). The touchscreen was positioned in front of the participant, centered along the body midline. In both experiments, the red square indicates the initial hand location of the right index finger (start), the white dot indicates the object and the green dot indicates the position (goal) where the participant had to bring the object using a swipe movement. In Experiment 1 (left panel), objects could appear in four directions (right, far, left, near from participant’s body) and two distances (small, large) from the center. The dotted line corresponds to the limits of the workspace during the Instructed Tablet task. Experiment 2 (right panel) consisted only of the Free task with the difference from Experiment 1 that the start, object and goal were arranged along the same horizontal line. Absolute and relative positions of start, object and goal could be varied independently.

Experiment 1 involved 10 participants (average age 21 years, 10 female). Objects appeared in one of four possible directions from the central start location (right, far, left, near from the participant’s body) at small (8 cm) or large (12 cm) distances (see Figure 1, left panel). The start location of the hand was near the center of the screen 20 cm from the body. Participants were asked to drag the object into the goal, which was in the same position as the start. Task difficulty was manipulated by the size of the goal, which had a radius of either 1 or 2 cm. We manipulated the flexibility of the relationship between the object and the location where the finger touches the screen to begin the swiping movement (initial swipe location). To this end, we explicitly instructed participants to start the swiping movement on the object location (*Instructed Reach task*), or to start swiping without touching the object (*Instructed Tablet task*), or to freely choose where to start the swiping movement (*Free task*). In the Free task, the object could be controlled by positioning the index finger anywhere on the screen, so that participants could choose freely whether to put their finger onto the object or to use a spatial mismatch between the object and their finger. In the Instructed Tablet task, participants could only position their index finger within a limited range of the touchscreen (10 cm square in the center of the screen, dotted rectangle in Figure 1, left panel), enforcing a spatial mismatch between the finger and the object, that was situated outside the square, for the swiping movement. The perimeter of the virtual workspace on the touchscreen was shown at the beginning of the trial but it disappeared as soon as the finger was again put on the touch surface and the object location was locked to the finger position. In this way, the tablet-like perimeter limited only the positioning of the index finger and not the following movement to bring the object into the goal. If participants locked the object outside the workspace, an error message was shown on the screen and the trial was repeated. In the Instructed Reach task, participants could only start the swiping movement if they touched the screen directly on the object, i.e., within 2 or 4 cm from the object center.

In all tasks, participants were instructed to bring the object into the goal as fast and as accurately as possible. To avoid that the behavior imposed in the Instructed Tablet task influenced the performance in the Free task, the task order was fixed across participants (1st Free task, 2nd Instructed Tablet task, 3rd Instructed Reach task). Although all participants were experienced touch screen users, we familiarized them with the present touch screen and tasks during a practice session of 15 trials before the main experiment started. For each task in the main experiment 160 trials were collected, repeating the 16 conditions (4 object directions × 2 object distances × 2 goal sizes) 10 times in a randomized order.

### Experiment 2: Relative Coordinate System in Swiping Behavior

In the second experiment, we investigated the role of the (relative) locations of start, goal and object on the swiping strategy that we observed in the Free Task in Experiment 1. Twenty-nine right-handed participants (average age 22 years, 24 female) performed a one-dimensional version of the Free task presented in experiment 1. Object, start and goal locations were arranged along a virtual fronto-parallel line on the screen and their positions on the screen were varied independently: goal and start could appear in the center or 12 cm to the left or to the right from the center, in the same or in different locations (see Figure 1, right panel). The object could appear at the center, to the left or to the right at 6, 12, or 18 cm from the center, with the constraint to be in a different position than the start and the goal location. Because the size of the goal did not influence the behavior in Experiment 1, we kept the goal size fixed to a 2 cm radius. In total, Experiment 2 contained 48 conditions ([3 start locations × 3 goal locations × 7 object positions] − [15 excluded start-goal-object combinations]).

After a practice session of 10 trials, each condition was repeated four or eight times for each participant depending on time availability, for a total of 192 or 384 trials respectively (4 repetitions for 10 participants, 8 repetitions for 19 participants). The order of conditions was pseudo-random so that the same condition was not repeated in the next trial. The total duration of the experiment was between 30 min and 1 h, including time for instructions and small breaks during the experiment every 4 or 8 min, to allow participants to rest their arm.

### Analyses

Data analyses were performed in Matlab (The MathWorks). We recorded only trials that were completed within the 2 s of the trial duration. Among those, we analyzed only the trials in which participants did not leave the screen before completing the swipe movement (Percentage of excluded trials in Experiment 1: Free task: 9.7%; Tablet task: 13%, Reach Task: 4.6%; Percentage of excluded trials in Experiment 2: 9.4%).

**Figure 2:**
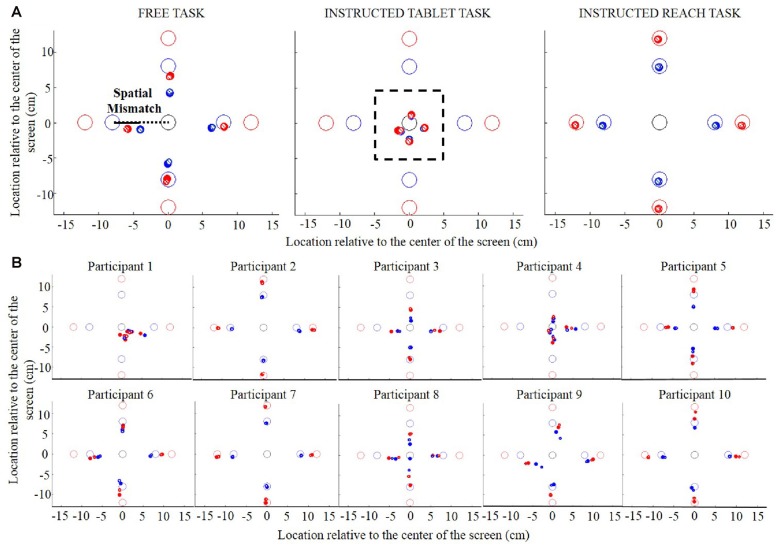
**(A)** Object location and initial swipe location for the Free task (left panel), the Instructed Tablet task (central panel) and the Instructed Reach task (right panel) in Experiment 1. The open circles represent the objects. The dots represent the initial swipe locations averaged across participants. Small striped and larger filled dots show initial swipe locations during trials with small and large goal sizes, respectively. The colors of the circles and the dots represent the object distance from the center (blue, small distance; red, large distance). The black dotted line indicates the line spanned by the start and the object and the black line indicates the spatial mismatch calculated as the difference between the location of the object and the initial swipe location along the dotted line. **(B)** Object location and initial swipe location in each participant for the Free task in Experiment 1.

In both experiments, for each participant we calculated the distance between the location of the object and the initial swipe location, which we refer to as the *spatial mismatch*. While the spatial mismatch can be defined as a 2D vector, its component orthogonal to the line connecting the object and start location was found negligible, and did not change the significance of the effects. We therefore calculated the spatial mismatch as the difference between the location of the object and the initial swipe location along the line spanned by start and object (see visualization of the spatial mismatch in Figure 2A, left panel). For objects in directions left and right from the start, positive and negative values of the spatial mismatch indicate initial swipe locations to the left and to the right of the object, respectively. That is, an “undershoot” relative to the object led to a positive spatial mismatch for the right object and to a negative spatial mismatch for the left object. Although this sign convention may seem counterintuitive for Experiment 1, we used it because it allows for the simplest description of the data in Experiment 2. Similarly, or far and near objects (only in Experiment 1), positive and negative values of the spatial mismatch indicate initial swipe locations nearer and further than the object, respectively.

In Experiment 1, we analyzed the (unsigned) size of the spatial mismatch by conducting repeated-measures analysis of variance (ANOVA) separately for the Free and the Instructed Tablet tasks. Specifically, we tested whether the spatial mismatch was influenced by goal size (small, large), object distance (small, large) and object direction (right, far, left, near the center of the screen). To avoid that the adopted sign convention created artificial differences between object distances, we performed the ANOVA on the absolute values of spatial mismatch.

Within-participants’ contrasts were run for object direction. To check whether the behavior changed throughout the experiment during these tasks, we conducted two separate ANOVAs on the spatial mismatch, separately for the first and last part of the experiment (first 5 repetitions vs. last 5 repetitions) with factors goal size, object distance and object direction. We tested whether error rates differed between tasks analyzing the number of error in each condition and in each task. Specifically, we conducted an ANOVA with factors Task (3 levels), Goal Size (2 levels), Object Distance (2 levels) and Object Directions (4 levels). To investigate the interaction between task and object direction, we conducted an ANOVA for each task.

In Experiment 2 a multiple linear regression was performed to quantify the effect of start, goal and object positions on the spatial mismatch, separately for each participant. To help interpret the results of this regression, the regression slopes were subjected to a principal component analysis (PCA).

## Results

We conducted two experiments in which participants had to make a swipe movement across a touchscreen to move an object from its initial location to a goal location on the display. In some conditions, participants could freely decide where to touch the screen to start the swipe, from touching the screen at the exact location of the object to anywhere else, exploiting the flexibility of object control when swiping over a touchscreen. By measuring the spatial difference (called spatial mismatch) between the object and the initial swipe location, we could identify the preferred behavior adopted by participants to interact with objects on touchscreens.

In Experiment 1, we tested the difference between allowing a flexible visuomotor mapping (the Free task), as typically allowed on a touchscreen, and two instructed visuomotor mappings (Instructed Tablet task and Instructed Reach task, see “Materials and Methods” section). In the Free task, participants were allowed to initiate their swiping movement anywhere on the screen. During the Instructed Tablet task, the size of the workspace was limited to a small area around the start, evoking an instructed visuomotor mapping by excluding the possibility to touch on the object. During the Instructed Reach Task, participants had to touch the screen at the location of the object in order to move it. If participants preferred to directly touch the object, as one needs to do in tasks with physical objects, the spatial mismatch between the object and the initial swipe location should be zero, even in the Free task. By contrast, if participants preferred another strategy, we should measure a spatial mismatch not only when it was instructed (i.e., in the Instructed Tablet task), but also in the Free task.

### Spatial Mismatch in Free Swiping

During the Instructed Tablet task (Figure 2A, central panel), in which objects (open circles) could not be reached because they were outside the workspace, we measured specific initial swipe locations (dots) for different object directions and distances (blue circles and dots, small distance; red circles and dots, large distance). Across participants, the initial swipe location for small and large distances did not differ. This can be observed by the overlapping blue and red dots in Figure 2A (central panel), suggesting that the initial swipe location was determined by object direction and not by the exact object location. In the Instructed Reach task, participants were explicitly asked to start the swiping movement on the object. As expected, they accurately reached the objects in this task, as indicated by the negligible spatial mismatch (Figure 2A, right panel). However, in the Free task, where participants were allowed to use the entire screen for their swipes, they showed a systematic mismatch between the object and the initial swipe location (Figure 2A, left panel; see Figure 2B for individual performance in the Free task). In this task, the spatial mismatch between the initial swipe location and object was not a fixed value, but it proportionally increased as the distance between the start and object increased. This scaling of the mismatch with the start-to-object distance was seen in all directions. Averaged across object directions and goal size, the proportion did not differ for the small vs. the large distance (0.36 vs. 0.35, respectively, *t* = 0.243, *p* = 0.81). Finally, this proportion was not influenced by the size of the goal, as shown by overlapping small striped and larger filled dots in all panels in Figure 2.

**Figure 3:**
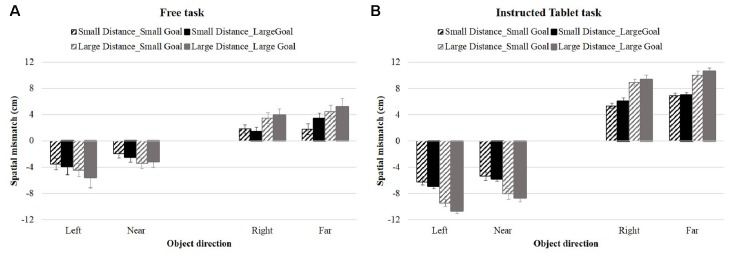
Spatial mismatch during Free task **(A)** and the Instructed Tablet task **(B)** for each object direction in Experiment 1. Black and gray histograms show the spatial mismatch for objects at small and large distances, respectively; striped and solid histograms show the mismatch during trials with small and large goal sizes. Error bars show the Standard Error.

Figure 3 shows the spatial mismatch between the object and the initial swipe position for the four object directions in the Free and Instructed Tablet tasks. For all four object directions, participants started the swipe between the start and objects locations (positive spatial mismatch for objects in the right and far directions and negative values for objects in the left and near directions). To interpret Figure 3 correctly, it is important to keep in mind that the spatial mismatch reflects the difference between the object location and the initial swipe location and not the distance of the initial swipe location from the center of the screen.

In both tasks, the spatial mismatch increased with object distance, as indicated by different black and gray lines, reflecting different spatial mismatches for objects at small and large distances, respectively. Participants started their swipes closer to the object if it was closer to the start/goal location. This behavior was modulated by the goal size only during the Tablet task, as indicated by a slightly larger spatial mismatch for small goal size (dotted line) compared to large goal size (solid line). Furthermore, the spatial mismatch was smaller for near objects and objects positioned to the right compared to objects far and left, both for objects at small (black lines) and large distances (gray lines). These observations were confirmed by the ANOVA’s showing significant main effects of object direction and object distance in both tasks and a main effect of goal size only in the Tablet task (see Table 1). Similar results were obtained when the data were separated into an initial and final phase of the experiment, where object distance and directions had significant effects in both phases (Free Task-Initial phase: object distance: *F*_(1,9)_ = 16.286, *p* = 0.003, object direction: *F*_(3,27)_ = 5.788, *p* = 0.021; Free Task-Final phase: object distance: *F*_(1,8)_ = 7.510, *p* = 0.025, object direction: *F*_(3,24)_ = 4.960, *p* = 0.045; Tablet task-Initial phase: object distance: *F*_(1,9)_ = 3119.307, *p* < 0.0001, object direction: *F*_(3,27)_ = 19.015, *p* < 0.0001; Tablet task-Final phase: object distance: *F*_(1,9)_ = 72.907, *p* < 0.0001, object direction: *F*_(3,27)_ = 10.685, *p* < 0.0001), while effects of goal size were significant only in the first part of the Tablet task (*F*_(1,9)_ = 10.155, *p* = 0.011). We tested whether the error rates differed between tasks using an ANOVA. We found a main effect of Task [*F*_(2,18)_ = 4.323, *p* = 0.029] and an interaction with object direction [*F*_(6,24)_ = 3.306, *p* = 0.008]. To explore this interaction, we conducted an ANOVA separately on each task and found that object direction was the only significant factor in the Free task (*F*_(1,9)_ = 4.253, *p* = 0.014), but not in the Tablet and Reach tasks.

**Table 1:** Results of the analysis of variance (ANOVA) on the spatial mismatch in Experiment 1.

	Free task		Tablet task	
	*F*	*p*	*F*	*p*
Goal size	0.665	0.436	16.242	**0.003**
Object distance	19.296	**0.002**	266.578	**<0.001**
Object direction	9.448	**<0.001**	16.477	**<0.001**
Goal size × Object distance	1.077	0.326	2.100	0.181
Goal size × Object direction	0.570	0.640	1.158	0.344
Object distance × direction	1.341	0.282	1.250	0.311
Goal size × object distance × object direction	1.188	0.333	0.799	0.505

*Bold indicates statistical significance*.

### Spatial Mismatch Depends on Relative Position of Start, Object and Goal

**Figure 4:**
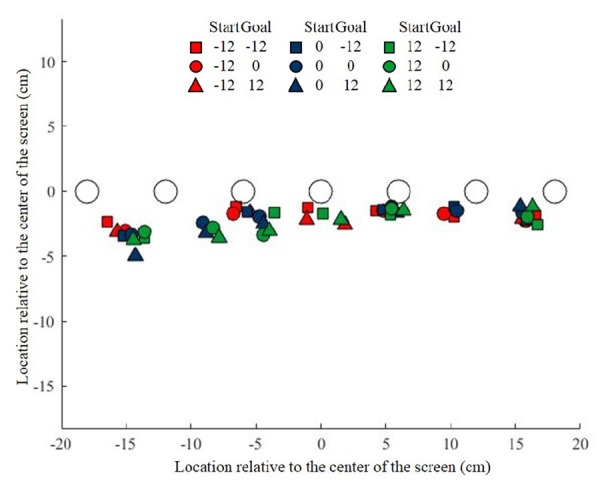
Object location and initial swipe location for the seven objects during Experiment 2. Each color corresponds to a specific start position, and each symbol correspond to a specific goal position, specified in the schematic legend above the figure.

In the previous experiment, we showed that participants exploit the flexibility provided in the Free task by selecting a visuomotor mapping that generates a systematic spatial mismatch between the object and the initial swipe position. In Experiment 2 we investigated in more detail how this spatial mismatch is determined. To this end, we presented the start, object and goal at different locations and tested to what extent each factor influences the spatial mismatch during the Free task. Figure 4 illustrates the initial swipe position average across participants for the seven objects during the nine combinations of Start and Goal positions. This figure shows that, like in Experiment 1, participants chose to use a spatial mismatch between their initial swipe position and the object.

**Figure 5:**
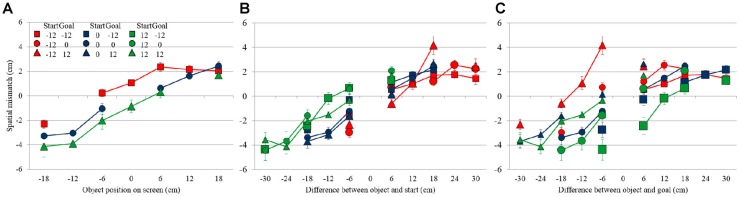
Spatial mismatch in Experiment 2, **(A)** as a function of object location on the screen in conditions where the start and goal were in the same location, **(B)** as a function of the object location relative to the start location **(C)** and relative to the goal location. Error bars show the Standard Error.

Figure 5A shows the average spatial mismatch as a function of object location on the screen. Data are shown only for conditions in which start and goal were at the same location, either in the center (blue circles connected by a blue line), or 12 cm to the left (red squares/line), or 12 cm to the right (green triangles/line) of the center. In these conditions, the hand moves first away from the start to initiate the swipe in the neighborhood of the object and then moves back toward the same location dragging the object into the goal. If the spatial mismatch depends only on the location of the object on the screen, the three lines should overlap, but they clearly do not. If the spatial mismatch depends only on the difference between the object and the start-goal location, we would expect the three response curves to be parallel, horizontally offset by 12 cm. Although there is some shift between the curves, the data does not completely conform to this hypothesis, so we performed a further analysis, factoring out goal and start location effects, to understand the observed mismatch pattern.

Figure 5B shows the spatial mismatch for all conditions (i.e., also including those in which the start and goal were at different locations) as a function of the location of the object relative to the start, showing that this rearranges the data into virtually a single response curve across the various conditions. In this figure, different colors correspond to different start locations, whereas different symbols correspond to different goal locations. If the spatial match is expressed as a function of the object relative to the goal (Figure 5C), there is still substantial dispersion in the data, suggesting that the mismatch is less strongly related to the location of object relative to the goal than to the location of object relative to the start.

To further investigate the contributions of object, start and goal location on the spatial mismatch, also at the single participant level, we considered the following linear model (Beurze et al., 2006):

(1) $$\text{Spatial mismatch}={\text{a}}_{0}+{\text{a}}_{\text{object}}*\text{object}+{\text{a}}_{\text{start}}*\text{start}+{\text{a}}_{\text{goal}}*\text{goal}$$

**Figure 6:**
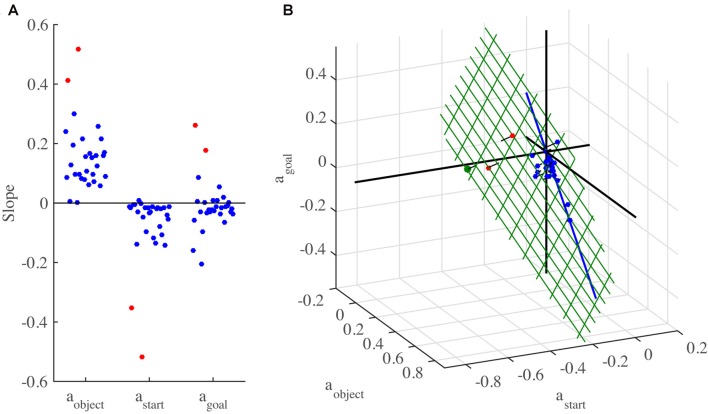
3D slopes of all individual participants in Experiment 2. **(A)** The three slopes plotted separately. The same participant order is used for all three slopes. The participants with divergent slopes are marked in red. **(B)** Slopes plotted in a 3D plot. The green grid represents the plane found by applying principal component analysis (PCA) to the 3D slopes. Orthogonal lines connect each point to the plane, to better indicate the 3D positions of the data points and to visualize how close the data are to the plane. The blue line shows the prediction of a preference not obscure the object by the finger. The green dot indicates the prediction of minimizing the expected total movement distance.

The constant coefficient a_0_ represents an offset, while a_object_, a_start_ and a_goal_ represent the slopes of the linear dependence of the spatial mismatch on the object, start and goal locations, respectively. We fit this model to the data of each individual participant. The offset a_0_ varied somewhat between participants, but was mostly not far from zero. The slopes of individual participants are shown in Figure 6A. These slopes display some systematic patterns. Whereas a_object_ is positive for all participants (mean: 0.155), a_start_ tends to be negative (mean: −0.071) and a_goal_ is positive for some participants, negative for others and averages close to zero (−0.010). The between-participant variability in all of these slopes is however large. Note that two participants (marked in red) behaved rather different than the others, as witnessed by their strong negative values for a_start_ and strong positive values for the other slopes.

To obtain a better understanding of the slopes, we made a 3D plot of the slopes of all participants in the space spanned by a_object_, a_start_ and a_goal_ (Figure 6B). Although it is difficult to see the exact 3D locations of the points, Figure 6B suggests that all data points were very close to a tilted plane in this space. To examine this further, we applied PCA to the 3D slopes. The first two principle components span a plane in this space (shown in green in Figure 6B; unit vector orthogonal to this plane: (a_object_, a_start_, a_goal_) = (0.51, 0.73 0.46)). The interpretation of this plane is that the 3D slopes do not occupy the whole 3D space, but for each participant, the three slopes approximately obey the relationship 0.51 * a_object_ + 0.73 * a_start_ + 0.46 * a_goal_ = 0. The first two principal components explain 97.9% of the variance in the 3D slopes, which confirms that the data points are close to this plane. This is not due to the two divergent participants (marked in red in Figure 6), as a PCA with these participants excluded produced almost the same plane (unit vector orthogonal to this plane: 0.46, 0.75 0.47), explaining 95.4% of the variance of the remaining 3D slopes.

To understand the meaning of this plane, one could formulate hypotheses about where participants decide to initiate the swipe movements, simulate the experiment following these hypotheses, and then fit equation 1 to the simulated data. For each hypothesis, this will lead to a point in the 3D space of Figure 6B.

Perhaps the simplest hypothesis is to start each swipe at the object, as this is how the task would be performed with real rather than virtual objects. According to this hypothesis, the Spatial mismatch is always zero, resulting in a 3D slope of (0 0 0), at the origin of Figure 6B, which is close to the 3D slope of only a couple of participants. Indeed, the fact that the slopes in Figure 6B span a whole plane suggests that three different factors were taken into account, as three points are needed to define a plane.

The second factor could be related to the first one. A disadvantage of starting the swipe movement exactly on top of the object is that the finger will obscure it from view, making it harder to visually track its position. Participants may therefore have chosen to start the swipe a bit off the object. To model this, we assumed that participants started the swipe at a point in between the start and object location, at a fixed proportion between them, i.e., at locations *w* * start + (1 − *w*) * object, with parameter *w* determining the proportion. The motivation for using a fixed proportion is that we found in Experiment 1 that the spatial mismatch was a fixed proportion (35%, corresponding to *w* = 0.35) of the Start-Object distance. This strategy results in a 3D slope of (*w* 0 −*w*), which is a line that falls almost perfectly in the plane (blue line in Figure 6B), regardless of the precise value of *w*. A preference to not obscure the object may thus be a factor that is taken into account.

A third hypothesis we considered is that participants moved as little as possible and did not even reach out to the object to start the swipe, i.e., they started their swipes at the start location. However, that would lead to a 3D slope of (1 −1 0), which is clearly outside the plane. Following a similar argument, the hypothesis that swiping movements were planned to end at the center of the touchscreen (so that the expected amplitude of the movement to the next start location is minimized) can be rejected (predicted 3D slope: 0 0 1).

We next considered the possibility that the expected total movement distance was minimized. In each trial, three movements are made, those from the start to the initial swipe location, the swipe movement itself, and the movement from the end location of the swipe to the start location of the next trial. The distance of the second of these is fixed, as it is determined by the task, but the amplitudes of the first and third movement depend on the chosen initial swipe location. We determined for each trial in the experiment the initial swipe location that minimized the expected total movement distance. We used the expected rather than the actual total distance, because the start location of the next trial was not known in advance. The expected total distance was found by averaging over the actual distances for all possible start locations of the next trial. If there was a range of initial swipe locations that minimized the expected total distance, we used the center of this range (using a different point within this range had little effect on the resulting slopes). This principle led to a predicted slope of (0.75 −0.72 0.28), which is very close to the plane (green dot in Figure 6B). This suggests that minimizing the expected total movement distance is the third factor that participants may have taken into account.

In summary, we found three factors that participants may have taken into account in deciding where to initiate their swipes: a preference to have the finger near the object, an aversion to obscure the object with the finger, and a preference to minimize the total expected movement distance. Each of these factors corresponds to a single 3D point in Figure 6B. If a participant took two or three of these factors into account, this would result in a point in between the points corresponding to the individual factors. Where exactly the point would fall depends on the weight given to each factor. By varying these weights, the whole plane in Figure 6B is spanned up. For instance, the two participants marked in red in Figure 6 gave a relatively high weight to minimizing the expected total movement distance, whereas all the others gave more weight to the other two factors.

## Discussion

We studied the visuomotor associations in touchscreen control by measuring where humans put their finger on a touchscreen to swipe an object from its initial location to a goal location. There is great flexibility in this association: the finger can be positioned anywhere on the screen to perform the swiping task. Nonetheless, we found that most participants choose to initiate their swipe at a position that systematically deviates from the object location. This deviation was not only present when participants were forced to use a spatial mismatch, because the objects were outside the workspace (Instructed Tablet task), but also when they were free to initiate their swipe anywhere on the touchscreen, including on the object (Free task). In a second experiment, we investigated how the initial swipe location was chosen in the Free task if the start location, object location and goal location could all vary independently. This experiment suggests that three factors are taken into account: a preference to have the finger near the object, an aversion to obscure the object with the finger, and a preference to minimize the total expected movement distance. The weight given to each factor varied across participants.

Previous studies on goal-directed reaching movements reported the distributions of end point errors in hand-centered, or eye-centered, instead of body-centered reference frames, suggesting that during reaching movements our brain computes a movement vector from the hand location to the object location (Gordon et al., 1994; Vindras et al., 1998, 2005). If the same mechanism as for reaching behavior were applied, the swiping movement would have started on the location of the object (no spatial mismatch). Given that participants in our Free task could have adopted default goal-directed reaching behavior, these results reveal a preference for a strategic motor behavior. Specifically, the distance between the hand and the object of the action is not the main variable to control during swiping, which differs from reaching movements where the minimal hand-object distance defines the accuracy of the reach.

Our results suggest that three factors are taken into account when deciding where to start a swiping movement. One of the factors is a preference to have the finger close to the object that has to be swiped to the goal, reflecting a tendency to perform the task in a similar way as with real objects. Furthermore, a direct contact between the hand and the object to manipulate increases performance compared to the same task when using a tool (Zheng and MacKenzie, 2007).

A second factor determining where to start a swiping movement is a preference not to obscure the object with the finger, by starting the swipe at a fixed proportion between the start and the object, allowing the use of online visual feedback control to move the object to the goal location.

One could argue that, in this perspective, it is more efficient to initiate the swipe on the object itself, because the finger and the object can both be controlled as a single entity, the position of which can be inferred from both vision and proprioception. If the use of the spatial mismatch was motivated only by the intent to create visibility of the object, one would expect that the spatial mismatch would be zero for objects larger than the one used here.

The third factor is a preference to minimize the expected total movement distance. Although the distance of the swipe itself was fixed and dictated by the task, the distances of the movements from the start location to the initial swipe location and from the final swipe location to the next start location depended on the chosen initial swipe location. Minimizing the expected total movement distance is consistent with many proposals of cost functions that are minimized for goal-directed reaches, such as energy expenditure (Alexander, 1997), endpoint variance of repeated reaches (Harris and Wolpert, 1998) and the changes in the joint torques required to make the reach (Uno et al., 1989).

From a control perspective, taking three different factors into account is more complicated than considering only a single one, but it has been shown before that our motor system can take into account multiple costs in the planning of arm movements (Berret et al., 2011; Morel et al., 2017). The weight given to the three different factors varied considerably across our participants. Our results do not allow us to determine on what basis individuals chose their weights, but we speculate that this could be related to their specific properties that are relevant for aspects of the task. For instance, a fatigued person may give a relatively large weight to minimizing the expected total movement distance, whereas a person with poor vision may give a large weight to good visibility of the object.

Does the present task have direct relevance to known touchscreen behaviors? Typically, we seem to interact with bigger objects on the screen than used here, such as swiping a globe. In such a case the visuomotor mapping is most flexible: the object can be manipulated from anywhere you touch it to swipe. The smaller objects used in the present study not only allow for a tangible way to measure the kinematic movement strategies, they also apply to real touch screen behavior. For example, our task mimics the situation of bringing (by a swipe) a particular location on a city map to the center of the screen.

Overall, in two experiments we showed that the visual-to-motor mapping between the location of the object and the location of the hand interacting with the object is different for swiping than reaching. The flexible relationship between the object location and initial swipe location provides a new approach to study the behavioral and neuronal substrates of visuomotor associations without the need to impose contextual rules to separate the object and the hand locations (Gail and Andersen, 2006; Gail et al., 2009). In particular, these results show that the motor system can successfully control objects even in absence of a spatial overlap between the hand and the objects to move. One could speculate that this behavior is facilitated by extending the representation of the hand peripersonal space, that is the space within hand-reaching distance. It has been shown that, both in humans and monkeys, the use of tools, like a rake, to interact with distant objects extends the peri-hand visual space to incorporate the tools (Iriki et al., 1996; Farnè and Làdavas, 2000). Also the computer mouse has a similar effect, extending the peripersonal space to the far space of the screen (Bassolino et al., 2010). While further experiments are needed to test the hypothesis that the hand’s peripersonal space is extended on a touch screen, our study suggest the possibility of developing different spatial representations in the real world and on touch screens.

## Author Contributions

SF contributed to the conception and design of the work, data collection, data analysis and interpretation and drafting the article, revising it and final approval of the version to be published. WPM and LPJS contributed to the conception and design of the work, data analysis and interpretation, revision of the article and final approval of the version to be published. RJB contributed to data analysis and interpretation, revision of the article and final approval of the version to be published.

## Conflict of Interest Statement

The authors declare that the research was conducted in the absence of any commercial or financial relationships that could be construed as a potential conflict of interest.
